# Imagine There Is No Plegia. Mental Motor Imagery Difficulties in Patients with Traumatic Spinal Cord Injury

**DOI:** 10.3389/fnins.2017.00689

**Published:** 2017-12-11

**Authors:** Aljoscha Thomschewski, Anja Ströhlein, Patrick B. Langthaler, Elisabeth Schmid, Jonas Potthoff, Peter Höller, Stefan Leis, Eugen Trinka, Yvonne Höller

**Affiliations:** ^1^Department of Neurology, Christian Doppler Medical Center, Paracelsus Medical University, Salzburg, Austria; ^2^Spinal Cord Injury and Tissue Regeneration Center Salzburg, Salzburg, Austria; ^3^Department of Psychology, Paris-Lodron University of Salzburg, Salzburg, Austria; ^4^Department of Mathematics, Paris-Lodron University of Salzburg, Salzburg, Austria; ^5^Center for Cognitive Neuroscience Salzburg, Salzburg, Austria

**Keywords:** spinal cord injury (SCI), motor imagery (MI), movement imagination, rehabilitation, neuroprostheses

## Abstract

In rehabilitation of patients with spinal cord injury (SCI), imagination of movement is a candidate tool to promote long-term recovery or to control futuristic neuroprostheses. However, little is known about the ability of patients with spinal cord injury to perform this task. It is likely that without the ability to effectively perform the movement, the imagination of movement is also problematic. We therefore examined, whether patients with SCI experience increased difficulties in motor imagery (MI) compared to healthy controls. We examined 7 male patients with traumatic spinal cord injury (aged 23–70 years, median 53) and 20 healthy controls (aged 21–54 years, median 30). All patients had incomplete SCI, with AIS (ASIA Impairment Scale) grades of C or D. All had cervical lesions, except one who had a thoracic injury level. Duration after injury ranged from 3 to 314 months. We performed the Movement Imagery Questionnaire Revised as well as the Beck Depression Inventory in all participants. The self-assessed ability of patients to visually imagine movements ranged from 7 to 36 (*Md* = 30) and tended to be decreased in comparison to healthy controls (ranged 16–49, *Md* = 42.5; *W* = 326.5, *p* = 0.055). Also, the self-assessed ability of patients to kinesthetically imagine movements (range = 7–35, *Md* = 31) differed significantly from the control group (range = 23–49, *Md* = 41; *W* = 337.5, *p* = 0.0047). Two patients yielded tendencies for depressive mood and they also reported most problems with movement imagination. Statistical analysis however did not confirm a general relationship between depressive mood and increased difficulty in MI across both groups. Patients with spinal cord injury seem to experience difficulties in imagining movements compared to healthy controls. This result might not only have implications for training and rehabilitation programs, but also for applications like brain-computer interfaces used to control neuroprostheses, which are often based on the brain signals exhibited during the imagination of movements.

## 1. Introduction

Motorimagery (MI) describes a variant of mental training that stands for the imagination of moving specific body parts (Schuster et al., 2011). As such it has been shown in athletes to not only decrease anxiety and enhance self-confidence but also to increase task performance (Martin et al., 1999; Munroe et al., 2000; Gregg et al., 2005). Furthermore, MI shares many similarities with the actual execution of movements with regard to the processes that take place in the central nervous system. For instance, it leads to similar activations of brain regions as planning, preparation and execution of movements (Decety, 1996; Grafton et al., 1996; Mellet et al., 1998), with the addition of activated areas required to inhibit an actual motor response (Deiber et al., 1998). Interestingly, imagined movements further seem to follow to some extent the same restrictions as executed movements, i.e., the pace of imagined movements is nearly the same as that of executed movements (Decety and Boisson, 1990; Decety and Jeannerod, 1996; Sirigu et al., 1996). These similarities lead to a general interest of using MI for several purposes such as training athletes in sports. Besides its beneficial role in sports training, MI has also been studied in clinical settings.

In several neurological conditions MI has been shown to have beneficial effects as an addition to rehabilitative therapies (Jackson et al., 2001; Braun et al., 2006; Dickstein and Deutsch, 2007; Schuster et al., 2011). Promoting long-term recovery by allowing to access the motor network without the need to actually move (Sharma et al., 2006), MI has also been considered for rehabilitation of patients suffering from SCI (Mulder, 2007). Searching the literature for motor imagery in relation to SCI reveals an increasing number of publications from 23 articles published until 2010 to already 40 articles since 2011 (source: pubmed accessed on 6th of October 2017; search terms “*motor imagery spinal cord injury*”). The majority of studies investigating MI in connection with SCI are either focusing on MI as a rehabilitative technique, or on brain signals elicited during MI.

Grangeon et al. (2010) reported that MI contributed to motor improvements equally as motor execution when integrated into physical therapy in one patient with quadriplegia. Cramer et al. (2007) reported positive effects of MI training in patients with SCI, however behavioral effects were only achieved in limbs that were not completely plegic. Still, even in paraplegic patients they reported brain activations during foot movement imagination that were similar to those observed in healthy controls. Focusing on these brain activations specific to MI in patients with SCI that have been reported in a variety of studies (Alkadhi et al., 2005; Müller-Putz et al., 2014; Foldes et al., 2017), there is another emerging field investigating a possible use of MI besides rehabilitation; recent studies investigated the possibility to use MI-elicited brain signals to control futuristic prostheses and devices via brain computer interfaces (BCI; e.g., Pfurtscheller et al., 2005; King et al., 2013; Rohm et al., 2013; Rupp et al., 2013).

Problems for the implementation of motor imagery arise due to a considerable variation between individuals in the ability to perform MI (Goss et al., 1986) and the resulting brain activations, suggesting that not every person is equally able to imagine movements (Pfurtscheller et al., 2006). Furthermore, we can differentiate visualization and, thus, spatial transformation from kinesthetic imagery (Hall et al., 1985). For interventional use, kinesthetic motor imagery (KMI) has a clear advantage over visual motor imagery (VMI), since cortico-motor excitability is affected by kinesthetic motor imagery only (Stinear et al., 2006). Concordantly it has also been suggested that KMI might be more suitable for BCI applications than VMI (Neuper et al., 2005).

The ability to imagine movements seems to determine the efficacy of MI training or its applications (Rodgers et al., 1991; Hall, 2001; Gregg et al., 2005) and this seems to especially hold true for KMI. The brain activation patterns elicited during KMI of professional athletics for instance differ considerably between sport experts and novices (Wei and Luo, 2010). In addition, Olsson (2012) reported findings from a patient with complete traumatic SCI who was an elite wheelchair athlete. Performing MI of a task that the patient was able to execute (wheelchair slalom) yielded brain activation in the pre-motor cortex. However, when performing a task that he was unable to execute (stair walking) this activation was absent. Concordantly, this pattern was reversed for healthy controls. These findings suggest a link between the ability to perform a movement and MI capabilities. Such a link could also account for lower classification accuracies in MI-based BCIs reported for patients with SCI in several studies (Pfurtscheller et al., 2009; Do et al., 2013; Blokland et al., 2014; Müller-Putz et al., 2014). Nevertheless, there are also opposite findings suggesting that the ability to execute a movement does not determine the ability to imagine it (Lotze and Halsband, 2006; Di Rienzo et al., 2014a).

In order to further investigate this matter it is necessary to not only consider indirect measures of MI ability, but also to investigate the subjective experience of patients performing MI. Therefore, we extended the above mentioned search in order to include additional search terms for motor imagery questionnaires and further searched all references in articles that were found to be of relevance for this study. Table 1 lists our analysis of the studies we identified reporting on systematic assessments of subjective motor imagery abilities in patients with traumatic SCI. Systematic in this context means, a quantitative assessment that allows for comparisons between subjects and groups.

**Table 1:** Studies systematically assessing motor imagery experience in patients with traumatic SCI.

**Study**	***n* (f)**	**Age**	**Time**	**c:th:l**	**c:i**	**HC**	**Measure**	**Outcome**	**Finding**
Alkadhi et al., 2005	8 (3)	31.3 (22–43)	32 (4–76)	0:-8-	8:0	8	Specific	Vividness	Correlation with fMRI activation; no comparison with HC
Di Rienzo et al., 2014b	1 (0)	23	12	1:0:0	1:0	1	KVIQ	K/VMI vividness	No differences
Di Rienzo et al., 2014c	6 (2)	18–55	>6	6:0:0	6:0	6	KVIQ	K/VMI vividness	No differences
Di Rienzo et al., 2015	4 (2)	27.5 (21–33)	14.5 (6–32)	4:0:0	4:0	4	KVIQ	K/VMI vividness	No differences
Fusco et al., 2016	11 (2)	37 (31–62)	103.8 (44–322)	11:0:0	6:5	13	VMIQ	K/VMI vividness	No differences
Grangeon et al., 2012	1 (0)	23	8	1:0:0	1:0	0	KVIQ	K/VMI vividness	↑ VMI vs. KMI vividness
Gustin et al., 2008^*^	15 (0)	41.6 (26–67)	12.8 (2–32)	0:15:0	15:0	0	VAS	MI difficulty	No effect for neuropathic pain; ↓ difficulty after training
Hotz-Boendermaker et al., 2008	9 (3)	34.8 (27–42)	117.3 (24–240)	0:7:2	9:0	12	VMIQ	K/VMI vividness	No differences
Mateo et al., 2015^*^	6 (2)	30.3 (18–40)	13.7 (6–30)	6:0:0	3:3	6	KVIQ	K/VMI vividness	Not analyzed
Moseley, 2007^*^	5 (0)	32.2 (24–45)	134.4 (60–240)	0:1:4	0:5	0	VAS	Vividness	Not analyzed
Roosink et al., 2016	9 (2)	52.7 (25–72)	80.8 (14–135)	3:5:1	6:3	0	KVIQ-10	K/VMI vividness	Comparable to HC sample from Malouin et al. (2007)
Scandola et al., 2017	47 (6)	41.5 (20–72)	155.7 (12–528)	25:16:6	24:23	24	VMIQ-R	K/VMI vividness	↓ First-person vividness in tetraplegic vs. HC; ↓ in affected body parts vs. not affected
Vuckovic et al., 2015	2 (0)	45 & 32	3 & 4	2:0:0	1:1	0	KVIQ	K/VMI vividness	Not analyzed

n(f), number of patients (female); time: time since injury in months; HC, number of healthy controls; c:t:ls, cervical: thoracic:lumbar; c:i, complete:incomplete; finding, if not specified otherwise refers to comparison with HC; fMRI, functional magnetic resonance imaging; KMI, kinesthetic motor imagery; VMI, visual motor imagery; KVIQ(−10), Kinesthetic and Visual Imagery Questionnaire (Malouin et al., 2007); VMIQ, Vividness of Movement Imagery Questionnaire (Isaac et al., 1986); VAS, visual analog scale; VMIQ-R, revised version of VMIQ (Scandola et al., 2017);

* *Sample may also contain non-traumatic patients; Alkadhi et al. (2005) used a Likert scale question to assess vividness*.

Only seven studies compared the subjective MI experience of SCI patients with findings in healthy subjects. However, six of these suggested no significant differences in kinesthetic and visual MI between SCI patients and healthy participants. Only Scandola et al. (2017) reported decreased vividness of MI in patients with tetraplegia. It is worth noting though, that only Gustin et al. (2008) explicitly asked their participants to indicate experienced difficulties with MI tasks, whereas all other studies assessed the vividness of perceived imaginations. However, Gustin et al. (2008) did not assess healthy subjects as well to compare the results between groups. Such a comparison would be of interest as experienced difficulties might bear important implications for the usage of MI in patients with SCI. The present study therefore aimed to determine whether visual and kinesthetic imagination of movements are perceived more difficult in patients with SCI than in healthy participants.

## 2. Methods

### 2.1. Ethics

Thestudy was approved by the local Ethics Committee (Ethics Commission Salzburg/Ethikkommission Land Salzburg; number E-Nr1541) and was designed according to the Declaration of Helsinki. Written informed consent was obtained from all participants prior to the assessments.

### 2.2. Subjects

Within a study conducted at the Department of Neurology, Paracelsus Medical University Salzburg, Austria, we asked seven patients with cervical or thoracic spinal cord injuries to fill in the Movement Imagery Questionnaire Revised (MIQ-RS; Gregg et al., 2010). As depression is a common comorbidity in patients with SCI we assessed also depressive tendencies using the Beck Depression Inventory II (BDI-II) as a control variable (Hautzinger et al., 2006). The BDI-II questionnaire was completed by all patients at the beginning of the study. The MIQ-RS was given to the patients and they were asked to complete it at a quiet moment between additional experimental sessions that were not part of the presented analysis. These comprised magnetic resonance imaging and electroencephalography, as well as repetitive transcranial magnetic stimulation in some cases.

We assessed 7 men aged between 23 and 70 years (*Md* = 53) with traumatic SCI. The extent of motor and sensory impairment was classified using the International Standards for Neurological Classification of Spinal Cord Injury (ISNCSCI; Kirshblum et al., 2011). A detailed description of the included patient group is given in Table 2. In addition, we asked 20 healthy participants to take part in an online questionnaire containing both the MIQ-RS and the BDI-II. All of these healthy controls were also men and aged between 21 and 54 years (*Md* = 30). None of them reported any motor or sensory impairments.

**Table 2:** Characteristics of the patient group.

**Patient**	**Age (years)**	**AIS grade**	**Level of injury**	**Time since injury (months)**
1	53	D	C4	3
2	44	D	C7	216
3	48	D	C6	314
4	70	D	Th8	13
5	60	C	C4	204
6	23	C	C5	48
7	65	C	C4	19

*AIS, ASIA Impairment Scale*.

### 2.3. Materials

The MIQ-RS contains two scales of seven items each, assessing the ability to imagine how the execution of a specific movement would feel like (kinesthetic imagery scale, KIS) and the ability to imagine how a certain movement execution would look like (visual imagery score, VIS; Gregg et al., 2010). Each item consists of a statement describing a particular everyday movement (i.e., pulling a door handle, or grasping a glass). The participants are asked to perform the movement once and afterwards return to their starting positions. Then they are asked to visually or kinesthetically imagine the movement, after which each item is to be rated according to a 7-point Likert scale from 1 = “very hard to see/feel” to 7 = “very easy to see/feel.” Sum scores for both scales range between 7 and 49 with low values indicating increased difficulties to either visually or kinesthetically imagine movements. Since all participants were German native speakers, the MIQ-RS was independently translated into German by two native speaking members of our team and we constructed the final version using matching translations and in case of differences by consensus decision of the study team.

We used the German version of the BDI-II to assess the severity of depressive symptoms (Hautzinger et al., 2006). The BDI-II contains 21-items, each of which is formulated as a question asking the participant to indicate the occurrence of certain moods or types of behavior within the last weeks (i.e., how often one cries, whether one has difficulties concentrating). Each item gives a number of possible answers that are coded from 0 to 3, leading to a sum score between 0 and 63. A higher score indicates more severe depressive symptoms or a stronger tendency toward depression. It has been shown that the BDI reliably assesses depressive tendencies in patients with SCI (Judd et al., 1991) and it is still widely used in clinical and scientific contexts (Kennedy et al., 2016; Barbonetti et al., 2017).

### 2.4. Statistical analysis

We performed two Wilcoxon-Mann-Whitney tests for non-parametric independent samples to compare the two variables of interest, the VIS-score and the KIS-score, between patients and healthy subjects. Two additional tests were calculated to compare the variables age and BDI-II scores between the two groups. Additionally, we calculated the correlations using Kendall's Tau between VIS and KIS scores and the two variables BDI-II scores and age. We used exact test statistics and corrected the *p*-values for multiple comparisons using the Holm-Bonferroni method (Holm, 1979). The resulting *p*-values, being already corrected, were then interpreted using a threshold of *p* = 0.05. For statistical analysis we used the R-Environment (R Version 3.4.1; R Core Team, 2017), the package “Kendall” (McLeod, 2011), and a slightly updated version of the package “rankFD” (Konietschke et al., 2016).

## 3. Results

**Figure 1:**
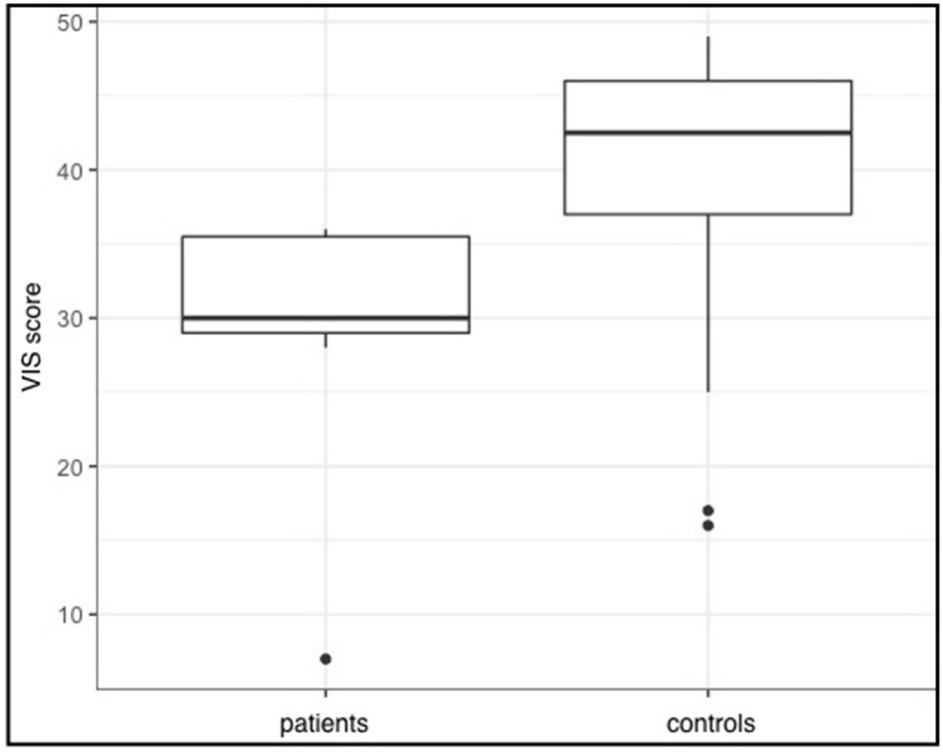
VIS sum scores for both groups. Given are the group medians as well as the first and third quartiles (represented by the hinges). Whiskers extend to higher and smaller values within 1.5 times the inter-quartile range from the hinges.

**Figure 2:**
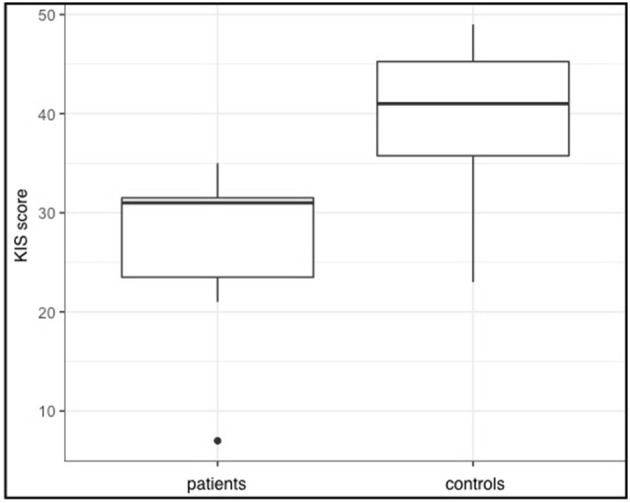
KIS sum scores for both groups. Given are the group medians as well as the first and third quartiles (represented by the hinges). Whiskers extend to higher and smaller values within 1.5 times the inter-quartile range from the hinges.

VIS scores of patients were lower (range = 7–36, *Md* = 30) than those obtained of healthy participants (range = 16–49, *Md* = 42.5; *W* = 326.5, *p* = 0.055), suggesting a trend for visual imagination of movements to be more difficult for patients with SCI. With regard to the kinesthetic imagination statistical analysis even suggested a greater difference between the groups, which was also statistically significant (*W* = 337.5, *p* = 0.0047). Again, healthy participants rated the imagination to be easier (range = 23–49, *Md* = 41) than patients with SCI (range = 7–35, *Md* = 31). The differences in VIS and KIS scores between the two groups are also depicted in Figures 1, 2. It seems plausible that patient 7 had a great impact on the statistical results. However, we used rank tests which are considered to be robust against outliers. Furthermore, we calculated the same tests again, without patient 7 and still found a significant difference between KIS scores of both groups (*W* = 317.5, *p* = 0.017). The tendency for lower VIS scores in patients however was not detected anymore (*W* = 306.5, *p* = 0.166). Therefore, the impact of patient 7 cannot be considered solely responsible for the differences found.

**Figure 3:**
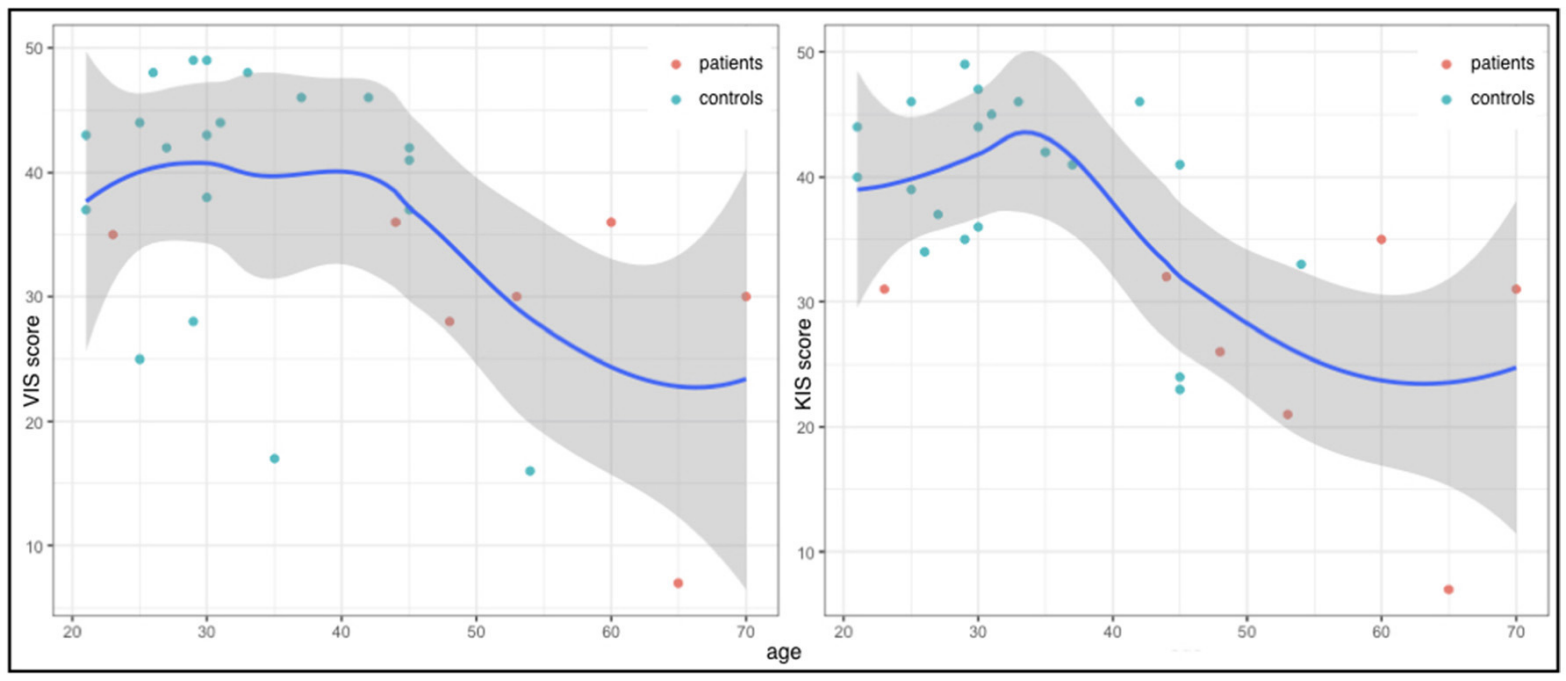
VIS (y-axis left) and KIS (y-axis right) scores and age (x-axes) for each subject of both groups as well as the general LOESS curves.

Furthermore, statistical analysis suggested a difference in age between the two groups with the patient group being older, though this difference was not significant after correction for multiple comparisons (*W* = 234, *p* = 0.055). Correlations between age and the two scores of interest revealed mild negative correlations suggesting greater difficulties in movement imagination in older participants (Figure 3). This relationship seemed to be more prominent for kinesthetic imagination (τ = −0.35, *p* = 0.0676) than for visual imagination (τ = −0.28, *p* = 0.1564).

**Figure 4:**
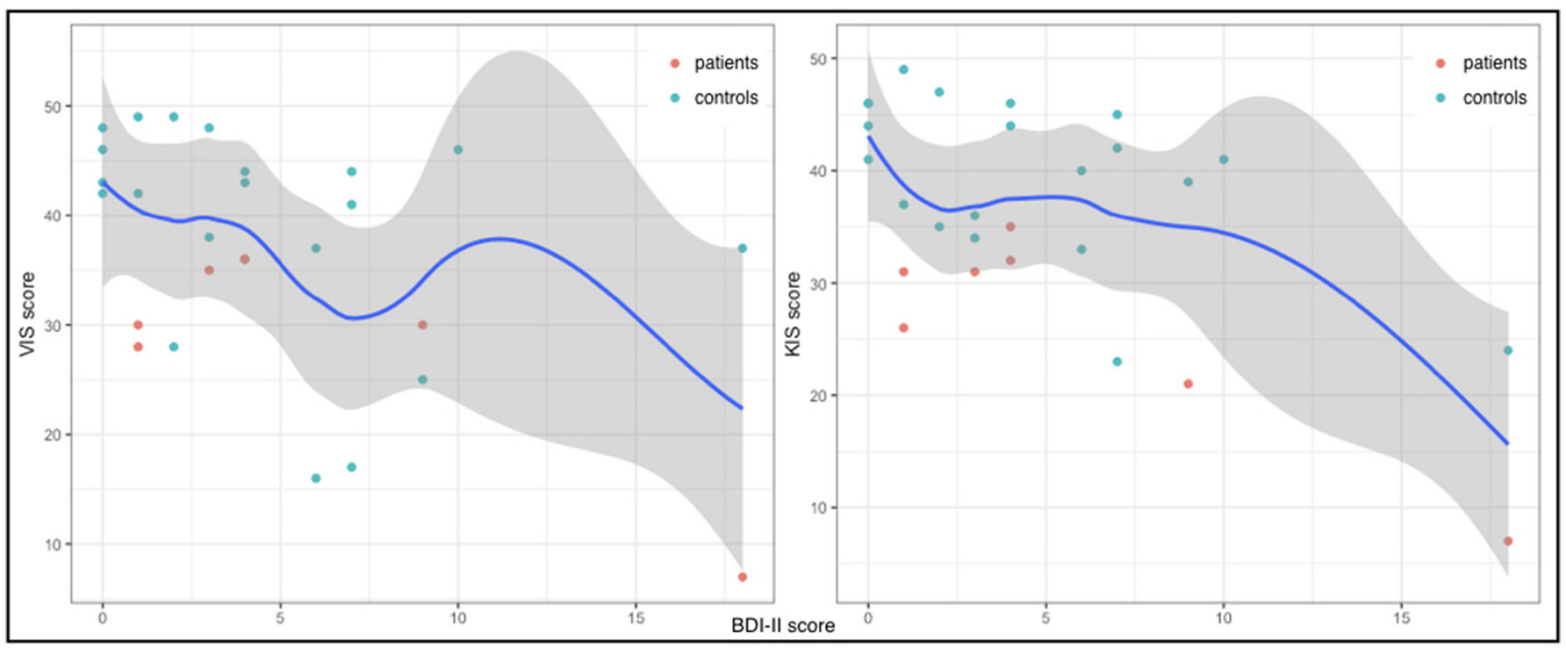
VIS (y-axis left) and KIS (y-axis right) and BDI-II scores (x-axes) for each subject of both groups as well as the general LOESS curves.

Patients and healthy controls did not differ with respect to their depressive tendencies as assessed using the BDI-II (*W* = 272, *p* = 0.6707). However, in both groups there were outliers (Figure 4). Patient 1 yielded a score indicating a minimal depression and patient seven was mildly depressed according to the BDI-II results. These two patients furthermore yielded the lowest values in both VIS and KIS scores. All results for the patient group are given in Table 3. Also, three healthy controls yielded scores in the BDI-II that suggested minimal and mild depressive tendencies. However, only two of them also yielded lower MIQ scores in comparison to their group. Statistical analysis revealed low negative correlations of BDI-II scores and both VIS (τ = −0.28) and KIS scores (τ = −0.3), though neither of them was significant (*p* = 0.1564 for both correlations).

**Table 3:** Results of the patient group.

**Patient**	**VIS**	**KIS**	**BDI-II**
1	30	21	9
2	36	32	4
3	28	26	1
4	30	31	1
5	36	35	4
6	35	31	3
7	7	7	18

*VIS, visual imagery scale; KIS, kinesthetic imagery scale; BDI, Beck Depression Inventory*.

## 4. Discussion

This study aimed to assess whether patients with SCI experience greater difficulties imagining movements than healthy controls. Indeed, our patient group reported greater difficulties in kinesthetic as well as visual motor imagery. Here we want to outline some limitations of the presented methods and point to some considerations regarding our patient sample on the basis of the obtained results. Finally, we will discuss possible confounding variables that might explain our results and draw conclusions from the presented findings.

### 4.1. Limitations

The main limitation is the small patient group assessed in this study. This is a known problem when it comes to studies in this neurological population. This can also be seen in Table 1, revealing only small sample sizes throughout most investigations, stressing the need for larger studies involving samples from multiple centers. The MIQ-RS not being standardized and designed for this particular patient group is a second limitation. Though, it has been validated in other neurological conditions, such as stroke (Gregg et al., 2010), there are no prior findings regarding validity or reliability in patients with SCI. The fact that subjects are asked to perform a certain movement before imagining it might introduce a confounding error, as patients with SCI are not able to perform all movements. However, there is no neuropsychological assessment of MI ability, that has been validated in SCI patients so far. Larger samples are necessary to assess the reliability of such questionnaires in this specific neurological subgroup. Regarding the MIQ results, one could also argue for an introduced error as healthy controls participated in an online assessment whereas patients were assessed using the pen and paper questionnaire. We assume that there is no difference between the two assessment forms, as both are subjective and equally prone to manipulation by participants.

### 4.2. Patient sample

In this study we investigated seven individuals with SCI who greatly differed concerning their time since injury. Alterations in brain functionality due to SCI-induced plasticity could impact MI abilities (Di Rienzo et al., 2014a). It has been suggested that plasticity occurs shortly after injury (Brasil-Neto et al., 1993; Aguilar et al., 2010). A longitudinal study found that atrophy in the central motor cortex is established as early as after 2 months (Freund et al., 2013). With regard to our sample we have found several indications that plastic changes had already occurred; in a previous study we documented changed or absent motor potentials obtained using electroencephalography during motor imagery and motor execution in the same sample as in this study (Thomschewski et al., 2017), and we found more diffuse and decreased brain activations during MI seen in functional magnetic resonance imaging scans. On this background, the absence of a visible connection between decreased MI abilities and time since injury in our sample seems plausible. We encourage further research that examines fast changes in MI ability in the acute stage of SCI.

It has been reported that the level of lesion as well as completeness of the injury have an impact on MI abilities (Pfurtscheller et al., 2009; Scandola et al., 2017). With regard to completeness of injury our sample was rather homogenous, as all were incomplete with AIS grades C, or D. In addition, all but one patient had cervical lesions and patient 4 with a thoracic lesion did not yield striking differences in experienced MI difficulties. Thus, albeit a small sample size, our patient group's heterogeneity is not likely to have been a confounding factor. However, given this sample, any conclusions drawn from these results should not be extrapolated to female patients or patients with more severe SCI resulting in complete injuries.

### 4.3. Depression as a confounding factor

As reported, there is a low correlation between depressive tendencies and increased difficulties to perform MI in the patient group. This is not too surprising, as the MIQ-RS asks for the subjective difficulty of MI and depression itself is characterized by difficulties in managing everyday activities, as reflected by the questions posed in the BDI. Also, a negative impact of depression on MI ability has already been reported previously (Bennabi et al., 2014) and might be of special importance in patients with SCI, as depression is a common comorbidity (Elliott and Frank, 1996; Kraft and Dorstyn, 2015; Williams and Murray, 2015). Taking into account the low and nonsignificant correlations in our whole sample between BDI-II and MIQ-RS scores, this finding should be interpreted with caution. Furthermore, it is well known that depression negatively impacts performance in all kinds of tasks (Cohen et al., 1982; Bennabi et al., 2014; Rock et al., 2014), implying a general effect on task performances rather than a specific effect on MI tasks. Nonetheless, we cannot rule out the possibility that in SCI, the depression is closely linked to movement capacities, and thus also to MI.

### 4.4. Age as a confounding factor

Our two groups did differ with respect to their age. Statistical analysis also suggested a mild correlation between age and difficulties in MI, though this relationship was not statistically significant after correction for multiple comparisons. This observed tendency does fit to similar reports of an age-related decline in MI (Saimpont et al., 2009; Malouin et al., 2010; Personnier et al., 2010). However, there are also contradicting findings in young individuals showing that MI capabilities increase at least in young ages (Hoyek et al., 2009). Given previous literature and the difference in age between our two groups, age on the one hand might very well contribute to the differences in MI difficulties between our groups. On the other hand, our findings also suggest that age does not solely explain the differences. Despite age as a possible contributing factor, our results suggest that motor impairments are likely to cause difficulties in imagining movement execution.

### 4.5. Conclusion

In summary, we found that our patients with SCI have more difficulties in imagining movements both visually and kinesthetically. This is likely to be at least partially caused by motor impairments and deafferentation, and should be kept in mind when implementing MI in rehabilitation or BCI-applications in this patient group. However, long-term effects of MI training probably help to overcome these experienced troubles, as has been suggested by Gustin et al. (2008). Furthermore, it is important to note, that this does not mean that the ability to perform MI is impaired, as it has been reported that MI vividness in patients with SCI is comparable to that of healthy subjects (Hotz-Boendermaker et al., 2008; Di Rienzo et al., 2014b,c, 2015; Fusco et al., 2016; Roosink et al., 2016). Additionally, we investigated visual and kinesthetic MI and it is possible that MI strategies may change after SCI (Fiori et al., 2014). Depressive tendencies and age might represent further contributing factors of increased difficulties in performing MI. In order to gain more insights into these issues, we suggest to assess subjectively perceived capabilities to perform MI in larger studies. Results obtained from a larger number of patients might give relevant insights on the exact nature of possible problems in MI performances experienced by patients with SCI and in further consequence have important implications on the incorporation of MI in rehabilitation, BCI applications, and neuroprostheses.

## Author contributions

AT and YH wrote the first draft of the manuscript. AT, JP, and PH collected the data from patients with SCI. AS and ES collected data from healthy participants. ES interpreted the BDI-II results. JP and SL translated the MIQ-RS. PL conducted the statistical analysis of the data. SL recruited patients for the study. SL and ET supervised the work in clinical respects and contributed ideas. All authors have read and revised the manuscript, which was coordinated by AT, who also produced the final draft for submission.

### Conflict of interest statement

The authors declare that the research was conducted in the absence of any commercial or financial relationships that could be construed as a potential conflict of interest.
